# Risk analysis of use of different classes of antidepressants on subsequent dementia: A nationwide cohort study in Taiwan

**DOI:** 10.1371/journal.pone.0175187

**Published:** 2017-04-06

**Authors:** Chee-Kin Then, Nai-Fang Chi, Kuo-Hsuan Chung, Lynn Kuo, Kao-Hui Liu, Chaur-Jong Hu, Shing-Chuan Shen, Yen-Kuang Lin

**Affiliations:** 1 School of Medicine, College of Medicine, Taipei Medical University, Taipei, Taiwan; 2 Graduate Institute of Medical Sciences, College of Medicine, Taipei Medical University, Taipei, Taiwan; 3 Department of Neurology, Shuang Ho Hospital, College of Medicine, Taipei Medical University, Taipei, Taiwan; 4 Graduate Institute of Clinical Medicine, College of Medicine, Taipei Medical University, Taipei, Taiwan; 5 Department of Psychiatry and Psychiatric Research Center, Taipei Medical University Hospital, Taipei, Taiwan; 6 Department of Psychiatry, School of Medicine, College of Medicine, Taipei Medical University, Taipei, Taiwan; 7 Department of Statistics, University of Connecticut, Storrs, Connecticut, USA; 8 Department of Dermatology, Shuang Ho Hospital, Taipei Medical University, Taipei, Taiwan; 9 Department of Pharmacology, College of Medicine, National Taiwan University, Taipei, Taiwan; 10 Biostatistics Center, Taipei Medical University, Taipei, Taiwan; University of British Columbia, CANADA

## Abstract

Depression and dementia are common mental health problems and are associated in several ways. Early-life depression is associated with increased risk of later life dementia, and depression can present as a preclinical symptom or consequence of dementia. Despite the plausible relationship between these two clinical entities, the potential association between antidepressant medication and dementia has rarely been investigated. We conducted a 9-year retrospective analysis of Taiwan’s National Health Insurance Research Database (NHIRD), enrolling 5819 cases who had received prescriptions of antidepressants between 2003 and 2006, and 23,276 (with ratio of 1:4) age, sex, and index date-matched controls. The hazard ratio (HR) of dementia among antidepressant users with depression was 2.42 (95% confidence interval (CI): 1.15–5.10), for those without depression was 4.05 (95% CI: 3.19–5.15), compared to antidepressant non-users respectively. Among the 6 classes of common antidepressants used in Taiwan, the adjusted HRs were 3.66 (95% CI: 2.62–5.09) for SSRIs, 4.73 (95% CI: 2.54–8.80) for SNRI, 3.26 (95% CI: 2.30–4.63) for TCAs, 6.62 (95% CI: 3.34–13.13) for TeCA, 4.94 (95% CI: 2.17–11.24) for MAOI, and 4.48 (95% CI: 3.13–6.40) for SARI. Furthermore, the multivariate analysis result showed that the adjusted HRs of cumulative defined daily doses (cDDDs) were 3.74 (95% CI: 2.91–4.82), 3.73 (95% CI: 2.39–5.80) and 5.22 (95% CI: 3.35–8.14) for those who had cDDDs of <90, 90–180 and >180 compared to those who had taken no antidepressant medication. This is a retrospective study based on secondary data, hence, we could not claim causality between antidepressant medication and dementia. However, a potential association between antidepressant and occurrence of dementia after controlling for the status of depression was observed. Lack of patients’ data about smoking status and body mass index in NHIRD, which are considered related to dementia, was also a limitation in this study. In this study, we concluded that antidepressant medication is a potential risk factor for dementia, independent from any effect of depression itself.

## Introduction

Dementia is a neurocognitive disorder characterized by cognitive impairment, mainly in elderly populations, and it leads to huge economic burdens around the world. Sun et al. reported that in Taiwan, the age-adjusted prevalence of all-cause cognitive impairment, which includes mild cognitive impairment (MCI) or very mild dementia (VMD), was 8.04% (95% confidence interval (CI): 7.47–8.61) [1]. Cognitive impairment is also frequently present in affective disorders such as depression [2], and the prevalence of depression in elderly was 6% [3, 4]. Depression and dementia often coexist and are associated in several ways. Overall, there is accumulating evidence indicating two consensuses that early-life depression is a risk factor for later life dementia, and depression can be a symptom or consequence of dementia [5–8]. Studies also reported that both entities share similar mechanisms of neurobiological alterations, especially white matter involvement, and proposed that they are comorbidities and share common risk factors or similar patterns of neuronal injury [8].

Regarding the complex interrelationship between the two clinical conditions, several studies supported a causative or risk factor hypothesis that depression is a risk factor for dementia [5, 9–14]. Byers et al. and Butters et al. suggested possible factors connecting depression to dementia, which include elevated β-amyloid plaques, glucocorticoid-induced hippocampal atrophy, generalized ischemic cerebrovascular disease, lack of nerve growth factors and inflammatory changes [9, 15]. Antidepressant medication is commonly used for treatment of depressive disorders, and it may also be prescribed for many other indications, including anxiety disorders, neuropathic pain, fatigue, sleep disorders, back pain, and headaches [16].

Antidepressants can be generally classified into several groups according to their proposed mechanisms of action, including tricyclic and tetracyclic antidepressants (TCAs and TeCAs), selective serotonin reuptake inhibitors (SSRIs), serotonin antagonist and reuptake inhibitors (SARIs), monoamine oxidase inhibitors (MAOIs), and selective norepinephrine reuptake inhibitors (SNRIs). In Taiwan, the prevalence of antidepressant use increased from 3.21% in 2000 to 4.63% in 2009 [17]. Although treating depression was believed to improve both mood and cognitive impairment, and animal studies showed neuroprotective effect of antidepressant [18], a large scale clinical trial of antidepressant failed to show efficacy of cognitive improvement [19]. Moreover, several studies mentioned that antidepressants may harm the patients in other aspects. The antidepressants could induce detrimental effects: (a) disruption of adaptive processes regulated by serotonin [20] in the aspects of emotion, attention [21], neuronal growth and death [22], astrocytic activity [23], electrolyte homeostasis [24], etc; (b) cytotoxicity to several human normal cells which include osteoclast, osteoblast [25], T-lymphocyte [26], and hepatocyte [27, 28]; and (c) increased neurological side effects: drowsiness, sedation [29], dizziness [30] and other extrapyramidal symptoms [31].

Despite the close relationship between depression and dementia, the potential association between antidepressant medication and dementia remains unclear. Based on the prior research as mentioned above, we hypothesized that antidepressant medication may actually increase the risk of dementia. To test this hypothesis, we used Taiwan’s National Health Insurance Research Database (NHIRD) to analyze the impacts of different classes of antidepressants and their cumulative dose-effects on the new diagnosis of dementia.

## Methods

### Data source

This is a retrospective population-based cohort study using Taiwan’s NHIRD [32]. The NHIRD was established in March 1995, and is one of the largest and most comprehensive population-based databases in the world; its completeness and accuracy are ensured by the Department of Health and the NHI Association of Taiwan. This NHI program consolidated 13 health insurance programs into a universal single-payer system to provide health care services to 99% of the population of Taiwan. In this study, we used a subset of NHIRD data containing 1 million beneficiaries of the National Health Insurance (NHI), randomly sampled from Taiwan’s general population of 23.74 million people. The database includes patients’ demographic characteristics, medical expenditures, details of prescription records (including drugs used, dosages, and days of supply dispensed), and three diagnoses with codes based on the International Classifications of Diseases, Ninth Revision, Clinical Modifications (ICD-9-CM).

### Study design and matching

The study cohort included 5819 cases with cumulative antidepressant use and 23,276 controls with no antidepressant use. These antidepressants and their Anatomical Therapeutic Chemical (ATC) codes consisted of SSRIs: Fluoxetine (N06AB03), Sertraline (N06AB06), Paroxetine (N06AB05), Fluvoxamine (N06AB08), Citalopram (N06AB04), and Escitalopram (N06AB10); an SNRI: Venlafaxine (N06AX16); TCAs: Imipramine (N06AA02), Amitriptyline (N06AA09), and Doxepin (N06AA12); a TeCA: Mirtazapine (N06AX11); a MAOI: Moclobemide (N06AG02); and a SARI: Trazodone (N06AX05). The study cases were defined as patients who had received antidepressants in ambulatory care between 2003 and 2006, and who complied with three conditions: (1) the interval between two continuous antidepressant-prescribed visits was less than 30 days; (2) the first and last antidepressant-prescribed visits were more than 30 days apart; and (3) they received only one type of antidepressant medication. Three groups of patients were excluded: (1) those aged over 80 years in 2015; (2) those with a history of dementia before receiving a prescription of antidepressants; (3) those of an undetermined sex. Based on the inclusion and exclusion criteria, 5819 cases were eligible for this study and their ages were ranged from 13 to 80. We defined the index date for each case as the date of the first drug prescription. The control group was sampled from the remaining patients in the NHIRD and consisted of patients with no antidepressant use between 2003 and 2006. With a 1:4 case-to-control matched schema in which controls were matched to cases according to age (±10 years), sex, and index date (±10 days), 23,276 patients were enrolled in the control group. The assumed average maintenance dose per day of a drug is called the defined daily dosage (DDD). Only drugs with an ATC code are assigned a DDD. Cumulative DDDs (cDDDs) were calculated using the following formula to compare any antidepressants based on the same standard:

$$\frac{\mathrm{total}\;\mathrm{amount}\;\mathrm{of}\;a\;\mathrm{drug}\;\mathrm{used}\;\mathrm{from}\;2003\;\mathrm{to}\;2006}{\mathrm{defined}\;\mathrm{daily}\;\mathrm{dosage}\;(\mathrm{DDD})}.$$

**Fig 1:**
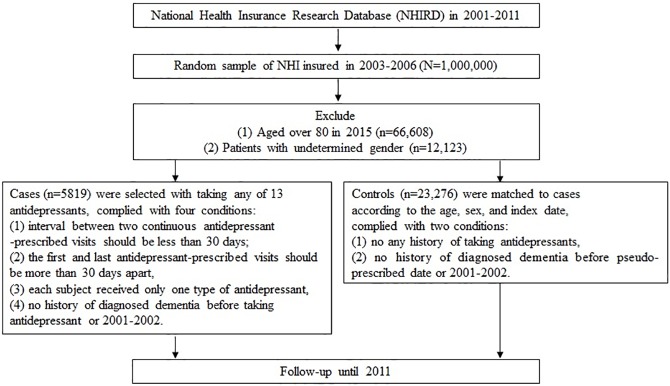
Flowchart of subjects selection.

cDDDs of each case were categorized into <90, 90–180 and ≥180 to reflect “less than three months”, “between three months and six months”, and “more than six months period” [33]. Antidepressant users were also categorized according to the potency of sedative and anticholinergic properties of the antidepressant medication being prescribed [34–36]. The sample collection process is depicted in Fig 1.

### Baseline confounding factors

Risk factors for each case and control were identified, including stroke (ICD-9-CM: 430–438), depression (ICD-9-CM: 296.2, 296.3, 300.4, 311.0), diabetes mellitus (DM; ICD-9-CM: 250), hypertension (ICD-9-CM: 401–405), hyperlipidemia (ICD-9-CM: 272), insomnia (ICD-9-CM: 307.41, 307.42, 307.49, 780.50, 780.52, 780.55, 780.56, 780.59), and anxiety (ICD-9-CM: 300.0–300.3, 300.5–300.9, 309.2–309.4, 309.81, 313.0). The baseline information was determined based on their medical history from 2001 to 2002 when the above mention ICD9 code occurs at least once. Scores of the Charlson comorbidity index (CCI) were applied to adjust baseline confounding factors weighted according to the mortality risk [37].

### Main outcome measures

In this study, follow-up periods ranged 1–9 years. In Taiwan, diagnoses of dementia are given by neurologists or psychiatrists, and are evaluated based on guidelines in Diagnostic and Statistical Manual of Mental Disorders, Fourth Edition (DSM-IV) or NINCDS-ADRDA Alzheimer's Criteria. The main outcome was confirmed by two repeated diagnoses of dementia (ICD-9-CM: 290, 294.1, and 331.0). Case and control patients were followed up from the index date until they received a principal diagnosis of senile dementia, uncomplicated (ICD-9-CM: 290.0), presenile dementia (ICD-9-CM: 290.1x), senile dementia with delusional or depressive features (ICD-9-CM: 290.2x), senile dementia with delirium (ICD-9-CM: 290.3x), arteriosclerotic dementia (ICD-9-CM: 290.4x), other specified senile psychotic conditions (ICD-9-CM: 290.8), unspecified senile psychotic condition (ICD-9-CM: 290.9), dementia in conditions classified elsewhere (ICD-9-CM: 294.1), or Alzheimer’s disease (ICD-9-CM: 331.0), or until 31 December 2011, whichever occurred first.

### Statistical analysis

Chi-square tests were used to compare distributions of demographic characteristics, including age group, sex, insurance amount categories, region, urbanicity, CCI score, and baseline risk factors, such as stroke, depression, DM, hypertension, hyperlipidemia, insomnia, and anxiety between the study and control groups. The cumulative incidence of dementia was estimated using the Kaplan-Meier method, and the differences in dementia-free distributions between case and control were determined using the log rank test. Cox proportional hazards regression models were used to compare the hazard ratios (HRs) for dementia of cases and controls along with their associated demographic characteristics, risk factors, CCI score, different classes of antidepressants, and cumulative DDDs. Proportional hazards assumption was examined by plotting the log(-log(survival function)) versus log of survival time. All analyses were performed using SAS 9.3 software (SAS Institute Inc., Cary, NC). In this study, each hypothesis was tested with two sided alternatives with its p-value reported. All significance levels were set to 0.05.

### Ethical approval

This study had appropriate Institutional Review Board (IRB) approval from Taipei Medical University (IRB approval number 201504060).

## Results

5819 antidepressant users and 23,276 antidepressant non-users were enrolled in this study. There were no significant differences between the case and control groups in terms of their age (M = 54.56 and 54.55 years, respectively) or sex distributions (males: 43.10% and 43.10%, respectively). The largest population who had received antidepressants in ambulatory care between 2003 and 2006 was a group of patients whose insurance amount was <NT$20,000. The prevalence of previous medical conditions that are considered to be possible risk factors for dementia, among cases were 6.14% for stroke, 16.65% for depression, 12.39% for DM, 19.38% for hypertension, 13.32% for hyperlipidemia, 19.68% for insomnia, and 23.68% for anxiety. All of the incidences of risk factors were significantly higher in cases than controls. However, both of the groups had comparable distributions of CCI score and the majority of subjects had the scores of less than or equal to 1. The mean of antidepressant cDDD of cases was 92.94 (Table 1).

**Table 1:** Comparison of demographic characteristics and risk factors between cases and controls.

		Study (N = 5819)		Control (N = 23,276)		P Value
		N	(%)	N	(%)	
Age, mean (SD) ^a^		54.56	(14.97)	54.55	(14.99)	.942
Sex (male) ^a^		2508	(43.10%)	10,032	(43.10%)	1.000
Insurance amount (NT$) ^b^^,^^c^						< .001
	≧40,000	597	(14.14%)	2955	(17.18%)	
	20,000–39,999	914	(21.64%)	4580	(26.62%)	
	<20,000	2712	(64.22%)	9670	(56.20%)	
Region ^b^^,^^c^						.916
	Northern	2565	(44.31%)	10,312	(44.51%)	
	Central	1414	(24.43%)	5740	(24.77%)	
	Southern	1639	(28.31%)	6622	(28.58%)	
	Eastern	171	(2.95%)	495	(2.14%)	
Urbanicity ^b^^,^^c^						.099
	1 (most urbanized)	1744	(33.23%)	7439	(35.12%)	
	2	1748	(33.31%)	6672	(31.50%)	
	3	823	(15.68%)	3621	(17.09%)	
	4 (least urbanized)	933	(17.78%)	3450	(16.29%)	
Risk factors ^b^						
	Stroke	357	(6.14%)	610	(2.62%)	< .001
	Depression	969	(16.65%)	434	(1.86%)	< .001
	Diabetes Mellitus	721	(12.39%)	2103	(9.04%)	< .001
	Hypertension	1128	(19.38%)	3903	(16.77%)	< .001
	Hyperlipidemia	775	(13.32%)	2514	(10.80%)	.040
	Insomnia	1145	(19.68%)	1876	(8.06%)	< .001
	Anxiety	1378	(23.68%)	2005	(8.61%)	< .001
CCI Score ^b^						< .001
	≦1	5191	(89.21%)	21,666	(93.08%)	
	2	362	(6.22%)	1023	(4.40%)	
	≧3	266	(4.57%)	587	(2.52%)	
Antidepressant cDDD, mean (SD)		92.94	(181.90)			
Antidepressant cDDD						
	<90	4153	(71.37%)			
	90–180	1020	(17.53%)			
	>180	646	(11.10%)			

^a^ *T* test.

^b^ Chi-square test.

^c^ Subjects for all levels do not sum up to original cases due to missing values.

**Fig 2:**
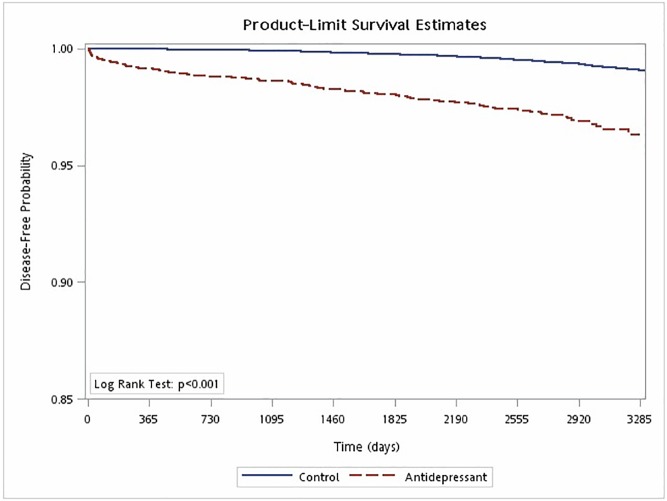
Kaplan-Meier estimates of study and control group patients who developed dementia.

The median follow-up times to dementia and their interquartile ranges for antidepressant users and nonuser were 6.74 [5.72–7.82] and 8.89 [8.75–8.93] years, respectively. The log-rank test indicated that cases were associated with a higher risk of dementia than controls (*p* < .001) (Fig 2). In terms of risk factors for dementia, age was positively correlated with an increased risk of dementia (Adjusted HR = 1.12 [95% CI: 1.11–1.14]). The incidence of dementia for antidepressant users was 2.68 per 100 persons, and the CI was 2.28–3.13 per 100 persons, while the incidence of dementia for non-antidepressant users was 0.86 per 100 persons, and the CI was 0.74–0.99 per 100 persons. We first applied the Cox proportional hazards model to the time to dementia since the first index date with the age, sex, insurance amount, region, urbanicity, depression, antidepressant usage, stroke, DM, hypertension, hyperlipidemia, depression, insomnia, anxiety, and CCI score as the predictors. All the predictors here are categorical including many binary indicators, except age. In order to examine whether the depression could be a potential confounder in evaluating the risk of antidepressant on dementia, we further added the interaction between depression and antidepressant medication to this regression model. Our results show no significant interaction was observed between these two entities (p = 0.193). That is, although the adjusted HR for antidepressant may seem slightly higher for patient without depression than that for patient with depression (4.05 [3.19–5.15] vs. 2.42 [1.15–5.10]), the difference in HRs is not significant. Moreover, we only list the results of the predictors excluding the last eight disease risk factors which we would like to control. We note that age (p < .001), insurance amount (p < .001), urbanicity (p = .017), CCI score (p = .033), depression (p = .011), and antidepressant usage (p < .001) are significant (Table 2). The appropriateness of the Cox proportional hazards model is supported by the plot in S1 Fig, where the log(-log(survival function)) versus log(survival time) are plotted for case and control groups separately.

**Table 2:** Risk factors examination for dementia using cox proportional hazards regression model.

		Event, n	Rate (95% CI), per 100 persons	Univariate HR [95% CI]	Multivariate HR [95% CI]	P Value
Age (years)				1.13 [1.12–1.15]	1.12 [1.11–1.14]**	< .001
Sex						.806
	Female	210/16,555	1.27 [1.10–1.45]	1.00 [Reference]	1.00 [Reference]	
	Male	146/12,540	1.16 [0.98–1.37]	0.93 [0.75–1.14]	0.97 [0.79–1.21]	
Insurance amount (NT$)						< .001
	≧40,000	17/3552	0.48 [0.28–0.77]	1.00 [Reference]	1.00 [Reference]	
	20,000–39,999	38/5494	0.69 [0.49–0.95]	1.45 [0.82–2.57]	1.41 [0.79–2.49]	
	<20,000	179/12,382	1.45 [1.24–1.67]	3.09 [1.88–5.08]	2.63 [1.60–4.34]**	
Region						.515
	Northern	138/12,877	1.07 [0.90–1.26]	1.00 [Reference]	1.00 [Reference]	
	Central	100/7154	1.40 [1.14–1.70]	1.30 [1.01–1.68]	1.22 [0.94–1.58]	
	Southern	105/8261	1.27 [1.04–1.54]	1.18 [0.92–1.53]	1.12 [0.87–1.44]	
	Eastern	9/666	1.35 [0.62–2.55]	1.28 [0.65–2.52]	1.15 [0.58–2.25]	
Urbanicity						.017
	1 (most urbanized)	81/9183	0.88 [0.70–1.10]	1.00 [Reference]	1.00 [Reference]	
	2	103/8420	1.22 [1.00–1.48]	1.39 [1.04–1.86]	1.37 [1.02–1.83]*	
	3	55/4444	1.24 [0.93–1.61]	1.41 [1.00–1.98]	1.44 [1.02–2.02]*	
	4 (least urbanized)	73/4383	1.67 [1.31–2.09]	1.90 [1.39–2.61]	1.65 [1.20–2.26]**	
CCI Score						.033
	≦1	340/28,770	1.18 [1.06–1.31]	1.00 [Reference]	1.00 [Reference]	
	2	12/278	4.32 [2.25–7.42]	3.78 [2.80–5.10]	1.51 [1.07–2.15]*	
	≧3	4/47	8.51 [2.37–20.38]	5.03 [3.62–6.99]	1.53 [1.02–2.28]*	
Depression						.011
	[Without]	316/27,692	1.14 [1.02–1.27]	1.00 [Reference]	1.00 [Reference]	
	[With]	40/1403	2.85 [2.04–3.86]	2.85 [2.05–3.96]	1.59 [1.11–2.27]*	
Antidepressant Usage						< .001
	[Without]	200/23,276	0.86 [0.74–0.99]	1.00 [Reference]	1.00 [Reference]	
	[With]	156/5819	2.68 [2.28–3.13]	5.03 [4.04–6.26]	3.89 [3.08–4.92]**	
Depression*Antidepressant						.193
Antidepressant effect (Depression = N)				5.08 [4.01–6.43]	4.05 [3.19–5.15]	
Antidepressant effect (Depression = Y)				2.27 [1.08–4.77]	2.42 [1.15–5.10]	

*:p<0.05

**: p<0.01.

The main outcome was diagnosis of dementia (ICD-9-CM: 290, 294.1), or Alzheimer’s disease (ICD-9-CM: 331.0). The adjusted HRs were calculated after controlling for stroke, diabetes mellitus, hypertension, hyperlipidemia, depression, insomnia, anxiety, and CCI score. HR is abbreviation for hazard ratio, CI is for confidence interval and CCI is for Charlson comorbidity index. Antidepressant effects are nested in Depression * Antidepressant effects were calculated for patients with depression (Y) and without depression (N).

To analyze the relative drug safety of antidepressants, we also compared risks for dementia of antidepressants according to their classes after adjusting for potential risk factors. A Cox regression model was conducted with different drug classes as the independent variable adjusted for stroke, diabetes mellitus, hypertension, hyperlipidemia, depression, insomnia, anxiety, and Charlson comorbidity index score. The adjusted HRs were 3.66 (95% CI: 2.62–5.09) for SSRIs, 4.73 (95% CI: 2.54–8.80) for SNRI, 3.26 (95% CI: 2.30–4.63) for TCAs, 6.62 (95% CI: 3.34–13.13) for TeCA, 4.94 (95% CI: 2.17–11.24) for MAOI, and 4.48 (95% CI: 3.13–6.40) for SARI. The TeCA group had the greatest hazard ratio for dementia, while the TCAs group and SSRIs group are the two groups which had the lowest incidence of dementia (Table 3).

**Table 3:** Risk of dementia with the use of antidepressants from different classes (reference = control).

Exposed to	Study (N = 5819)		Univariate HR [95% CI]	Multivariate HR [95% CI]
	N	(%)		
Selective serotonin reuptake inhibitors (SSRIs)	2371	(40.75%)	4.10 [2.99–5.62]	3.66 [2.62–5.09]**
Selective norepinephrine reuptake inhibitors (SNRI)	384	(6.60%)	5.35 [2.91–9.85]	4.73 [2.54–8.80]**
Tricyclic antidepressants (TCAs)	1583	(27.20%)	4.64 [3.29–6.56]	3.26 [2.30–4.63]**
Tetracyclic antidepressants (TeCA)	223	(4.00%)	8.29 [4.24–16.25]	6.62 [3.34–13.13]**
Monoamine oxidase inhibitors (MAOI)	155	(2.66%)	6.72 [2.97–15.13]	4.94 [2.17–11.24]**
Serotonin antagonist and reuptake inhibitors (SARI)	1103	(18.96%)	6.56 [4.63–9.29]	4.48 [3.13–6.40]**

*:p<0.05

**: p<0.01.

SSRIs include Fluoxetine, Citalopram, Escitalopram, Fluvoxamine, Sertraline, Paroxetine; SNRI includes Venlafaxine; TCAs include Amitriptyline, Imipramine, Doxepin; TeCA includes Mirtazapine; MAOI includes Moclobemide; SARI includes Trazodone. The main outcome was diagnosis of dementia (ICD-9-CM: 290, 294.1), or Alzheimer’s disease (ICD-9-CM: 331.0). The adjusted HRs were calculated after controlling for stroke, diabetes mellitus, hypertension, hyperlipidemia, depression, insomnia, anxiety, and Charlson comorbidity index score.

To investigate the cumulative dose-effect of antidepressants on dementia, we categorized cases into three groups according to their cDDDs of <90, 90–180, and >180. The multivariate analysis result showed that the adjusted HR of the cDDDs was 3.74 (95% CI: 2.91–4.82) for those who were <90 compared to the controls. The adjusted HR for those who have cDDDs between 90 to 180 appears to be similar to those who were <90 (3.73 vs. 3.74), which might be an indication that cDDD less than 180 being a relatively safe cut-off point. The adjusted HR for cDDDs >180 is 5.22 and is about 40% increased compared to those who have cDDDs <90. The association of various levels of cDDDs to the risk of developing dementia was examined using trend test. The result also indicated that there is an upward trend for developing dementia along with the increase of cDDD (p < .001). Furthermore, higher potency of sedative and anticholinergic properties of antidepressant medications is not correlated to higher hazard ratios of dementia (Table 4).

**Table 4:** Examination of the risk factors of dementia in terms of the antidepressant cDDD and its side effects using cox proportional hazards regression.

		Event, *n*	Rate (95% CI), per 100 persons	Univariate HR [95% CI]	Multivariate HR [95% CI]	P for trend
Antidepressant cDDD						< .001
	No use	200/23,276	0.86 [0.74–0.99]	1.00 [Reference]	1.00 [Reference]	
	<90	108/4153	2.60 [2.14–3.13]	4.89 [3.84–6.24]	3.74 [2.91–4.82]**	
	90–180	24/1020	2.35 [1.51–3.48]	4.45 [2.90–6.84]	3.73 [2.39–5.80]**	
	>180	24/646	3.72 [2.39–5.48]	6.69 [4.36–10.26]	5.22 [3.35–8.14]**	
Side effects of antidepressant medications–Sedation ^a^						.139
	0	32/1763	1.82 [1.24–2.55]	1.00 [Reference]	1.00 [Reference]	
	1+	30/992	3.02 [2.05–4.29]	1.64 [1.00–2.71]	1.56 [0.95–2.57]	
	3+	31/1250	2.48 [1.69–3.50]	1.34 [0.82–2.20]	1.04 [0.63–1.73]	
	4+	57/1659	3.44 [2.61–4.43]	1.86 [1.21–2.87]	1.47 [0.95–2.28]	
Side effects of antidepressant medications–Anticholinergic ^a^						.112
	0	85/3429	2.48 [1.98–3.06]	1.00 [Reference]	1.00 [Reference]	
	1+	25/652	3.83 [2.50–5.61]	1.57 [1.01–2.46]	1.60 [1.02–2.50]*	
	3+	31/1250	2.48 [1.69–3.50]	0.99 [0.66–1.49]	0.84 [0.56–1.28]	
	4+	9/333	2.70 [1.24–5.07]	1.05 [0.53–2.08]	0.93 [0.47–1.85]	

*:p<0.05

**: p<0.01.

^a^ Scale: 0 = none; 1+ = slight; 2+ = low; 3+ = moderate; 4+ = high.

About the potency of sedative property of antidepressants, 0 for Fluoxetine, Citalopram, Escitalopram, and Sertraline; 1+ for Fluvoxamine, Paroxetine, and Venlafaxine; 3+ for Imipramine and Doxepin; 4+ for Amitriptyline, Mirtazapine, and Trazodone. About the potency of anticholinergic property of antidepressants, 0 for Fluoxetine, Citalopram, Escitalopram, Sertraline, Fluvoxamine, Trazodone, and Venlafaxine; 1+ for Paroxetine and Mirtazapine; 2+ for Imipramine and Doxepin; 4+ for Amitriptyline.

The main outcome was diagnosis of dementia (ICD-9-CM: 290, 294.1), or Alzheimer’s disease (ICD-9-CM: 331.0). The adjusted HRs were calculated after controlling for stroke, diabetes mellitus, hypertension, hyperlipidemia, depression, insomnia, anxiety, and Charlson comorbidity index score.

In order to evaluate the magnitude of risk factors on the effect of dementia, we ran a Cox regression model where the eight risk factors consisted of stroke, diabetes mellitus, hypertension, hyperlipidemia, depression, insomnia, anxiety, and Charlson comorbidity index score were treated as independent variables. Adjusted hazard ratios of risk factors for dementia were 2.55 (95% CI: 1.86–3.49) for stroke, 1.58 (95% CI: 1.18–2.13) for diabetes mellitus, 2.27 (95% CI: 1.78–2.90) for hypertension, 1.27 (95% CI: 0.98–1.65) for hyperlipidemia, 1.59 (95% CI: 1.11–2.27) for depression, 1.64 (95% CI: 1.26–2.14) for insomnia, and 1.49 (95% CI: 1.14–1.94) for anxiety (Table 5). In addition, to assess the robustness of our results, we performed sensitivity analysis and conducted the Cox proportional hazards regression models on each risk factor as subgroups. In the subgroup analysis, age group, gender, stroke, diabetes mellitus, hypertension, hyperlipidemia, depression, insomnia, anxiety, and CCI score were included as confounding factors. For example, for male patients, their adjusted HR for developing dementia was 4.45 (95% CI: 3.11–6.37). The adjusted hazard ratios for developing dementia are 4.96 (95% CI: 3.05–8.07), 3.91 (95% CI: 2.55–6.01), and 3.65 (95% CI: 2.60–5.14) for the subgroups of insomnia patients, diabetes mellitus patients, and hypertension patients respectively. In general, the results confirmed a similar trend that proposing antidepressant medication would increase risk of dementia and it supports our conclusion, regardless of the age group, gender, baseline risk factors or CCI score (Table 6).

**Table 5:** Assessment of risk factors for dementia using cox proportional hazards regression model.

Risk Factor	Univariate HR [95% CI]	Multivariate HR [95% CI]
Stroke	6.22 [4.72–8.21]	2.55 [1.86–3.49]**
Diabetes Mellitus	3.51 [2.78–4.43]	1.58 [1.18–2.13]**
Hypertension	4.06 [3.29–5.00]	2.27 [1.78–2.90]**
Hyperlipidemia	2.85 [2.25–3.60]	1.27 [0.98–1.65]
Depression	2.85 [2.05–3.96]	1.59 [1.11–2.27]*
Insomnia	2.94 [2.31–3.74]	1.64 [1.26–2.14]**
Anxiety	2.69 [2.12–3.43]	1.49 [1.14–1.94]**
CCI Score (2 vs. ≦1)	3.78 [2.80–5.10]	1.51 [1.07–2.15]*
CCI Score (≧ 3 vs. ≦1)	5.03 [3.62–6.99]	1.53 [1.02–2.28]*

*:p<0.05

**: p<0.01.

The main outcome was diagnosis of dementia (ICD-9-CM: 290, 294.1), or Alzheimer’s disease (ICD-9-CM: 331.0). The adjusted HR for each risk factor was calculated after controlling for the remaining seven risk factors.

**Table 6:** Subgroup analysis of cox proportional hazard regressions of antidepressant associated with the incidence of dementia.

Subgroup		Univariate HR [95% CI]	Multivariate HR [95% CI]
Age group	≦44	3.20 [0.72–14.32]	2.47 [0.47–12.91]
	45–64	8.20 [4.53–14.85]	8.34 [4.45–15.61]**
	≧65	4.73 [3.73–6.01]	3.84 [2.98–4.94]**
Gender	[Female]	4.80 [3.60–6.41]	3.59 [2.64–4.88]**
	[Male]	5.37 [3.82–7.54]	4.45 [3.11–6.37]**
Stroke	[Without]	4.81 [3.78–6.13]	4.08 [3.16–5.28]**
	[With]	3.09 [1.82–5.24]	2.90 [1.67–5.06]**
Diabetes Mellitus	[Without]	4.73 [3.64–6.13]	3.90 [2.95–5.15]**
	[With]	4.71 [3.11–7.13]	3.91 [2.55–6.01]**
Hypertension	[Without]	4.97 [3.68–6.71]	4.09 [2.93–5.58]**
	[With]	4.52 [3.28–6.23]	3.65 [2.60–5.14]**
Hyperlipidemia	[Without]	4.94 [3.82–6.39]	4.04 [3.08–5.32]**
	[With]	4.54 [2.98–6.90]	3.51 [2.24–5.49]**
Depression	[Without]	5.12 [4.04–6.49]	4.07 [3.20–5.19]**
	[With]	2.19 [1.02–4.67]	2.54 [1.17–5.54]*
Insomnia	[Without]	4.45 [3.44–5.75]	3.55 [2.71–4.64]**
	[With]	4.50 [2.85–7.09]	4.96 [3.05–8.07]**
Anxiety	[Without]	4.70 [3.63–6.10]	3.92 [3.00–5.13]**
	[With]	3.76 [2.41–5.89]	3.67 [2.28–5.91]**
CCI score	≦1	4.87 [3.77–6.30]	4.07 [3.09–5.35]**
	2	3.87 [2.19–6.85]	2.99 [1.60–5.61]**
	≧3	3.88 [2.06–7.33]	3.79 [1.97–7.27]**

*:p<0.05

**: p<0.01.

The main outcome was diagnosis of dementia (ICD-9-CM: 290, 294.1), or Alzheimer’s disease (ICD-9-CM: 331.0). The adjusted HRs were calculated after controlling for stroke, diabetes mellitus, hypertension, hyperlipidemia, depression, insomnia, anxiety, and Charlson comorbidity index score, except for the targeted risk factor itself.

## Discussion

In this study, we found that cases who had taken antidepressants had a higher incidence of dementia relative to the controls. The TeCA group had the highest HR at 6.62 (95% CI: 3.34–13.13), while the TCAs group and SSRIs group had hazard ratios of 3.26 (95% CI: 2.30–4.63) and 3.66 (95% CI: 2.62–5.09), which indicates that they are the least dementia-associated antidepressant classes compared to the other 4 classes. In addition, we categorized patients into four groups according to their cDDDs of antidepressants, to estimate the total drug amount that patients were exposed. We found that cDDDs of antidepressants >180 were associated with higher incidences of dementia. This phenomenon once again supports the idea that antidepressants may be a risk factor in inducing dementia.

Antidepressants with higher sedative properties (e.g., mirtazapine) and anticholinergic properties (e.g., TCAs) have a higher hazard ratio of dementia in this study. To eliminate the effect of some antidepressants that might lead to an earlier dementia diagnosis due to sedative effects and to control for depression and anxiety as prodromal symptoms of dementia, we conducted a sensitivity analysis which excludes patients diagnosed with dementia within the first 5 years following the data of registration of antidepressant treatment. The results are similar to the one without exclusion (S2 Fig). Besides that, we also evaluated the HRs of dementia according to the potency of antidepressants of sedative and anticholinergic properties [34–36]. We observed that there is no association between the side effects of antidepressant medications and risk of dementia (Table 4). One of the reasons is that medication-induced dementia, a reversible dementia, would be ruled out during making diagnosis of dementia. Furthermore, we only selected cases who only used one type of antidepressant to eliminate the confounding of drug-drug interaction.

**Fig 3:**
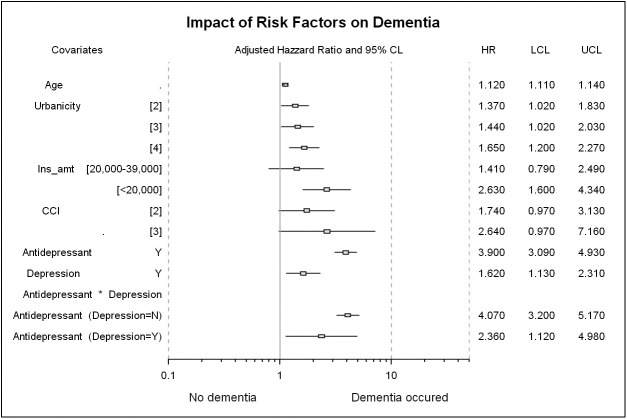
Forest plot of factors associated with the incidence of dementia.

In the pathogenesis of dementia, both ideas that mention depression as a risk factor for dementia and depression as a prodromal symptom remain controversial. It is difficult to clarify the causal relation between depression and dementia, and findings in the previous literature are inconsistent on these issues [10, 38, 39]. We found that dementia is a possible pathological consequence since patients with depression had higher incidence of dementia (HR = 1.59, 95% CI: 1.11–2.27) (Table 5). This finding is consistent with previous studies that identified depression as one of the risk factors of dementia [9, 10]. Considering the potential confounding effect of depression on dementia in this study, we first examined the interaction effect between depression status and antidepressant usage and then tested the antidepressant effect for depression and non-depression groups. The adjusted HR of dementia in depression group was 2.42 (95% CI: 1.15–5.10), while for non-depression group, it was 4.05 (95% CI: 3.19–5.15) (Table 2). Our result shows that antidepressant users had higher hazard ratio of dementia, which indicated that antidepressant medication is a potential risk factor for dementia and is independent of depression and other well-known risk factors, such as age, CCI score, stroke, diabetes, hypertension, hyperlipidemia, insomnia, and anxiety (Fig 3). Again, this result suggested that dementia may be an iatrogenic result of taking antidepressant medication, besides the pathogenic consequence of depression.

Subjects who are over 80 years old had higher proportion of risk factors and this may worsen the confounding of these baseline factors to our analysis in the study. Besides that, life expectancy in Taiwan is 80 years old and this subgroup may not have sufficient duration time for follow-up. Hence, the subgroup who exceeds 80 years old was excluded in this study. In addition, the reason that we set antidepressant users who had been prescribed at least 30 days is because depressive symptoms get improved within this period of time [36] and this criterion is widely accepted in other studies [40–42].

Patients in the SSRIs group had low HR for dementia, so we considered SSRIs to be lesser associated with dementia compared to the others. In addition, several studies supported our findings from a neurobiological view, which mentioned that Fluoxetine, one of the SSRIs, plays an important role in neuroprotection. Malberg et al. demonstrated that Fluoxetine can rescue the effect after exposure to inescapable shock (IS), which results in a state of behavioral despair and reduction of hippocampal cell proliferation [43]. Chen et al. revealed that Fluoxetine, either alone or in combination, can enhance hippocampal neurogenesis in long-term depression via a mechanism of its antidepressant action [44].

Our study reported 16.65% of antidepressant users have been diagnosed with depression, which is slightly lower than result presented by Chi-Shin Wu et al. in 2012 [17]. Such a low ratio of depression among antidepressant users is due to several reasons. In Taiwan, patients would only be coded for major depressive disorder (ICD-9-CM: 296.2, 296.3), dysthymic disorder (300.4), or depressive disorder, not otherwise specified (311.0) by psychiatrists if their symptoms meet the DSM-IV criteria. Besides that, the indications for antidepressant usage have been extended for various anxiety/panic disorders, not only for mood disorders [45]. Furthermore, antidepressants are widely prescribed for the other clinical accepted off-label uses include migraine [46], back pain [47], insomnia [48], etc. For instance, amitriptyline, one of the tricyclic antidepressants, is the most common treatment strategy for neurologic pain and fibromyalgia in many guidelines [49, 50].

Consistent with our results, Hsiao et al. reported that a higher ratio of dementia cases had a history of taking antidepressants (11.41%) compared to control (4.88%) (*p* < .001) [51]. That is, they established a solid association between the use of antidepressants and dementia. As mentioned previously, antidepressants are tightly related to depression, and depression is a well-known risk factor for dementia; but the effect between antidepressant medication or depression on increasing the risk of dementia is still unclear. We integrated several lines of evidence in previous studies, including (a) a study using Taiwan’s NHIRD which showed that populations with a dementia diagnosis had a higher ratio of taking antidepressants [51]; (b) antidepressant medication causes severe neurological side effects, which reflect antidepressants interfering with brain function, possibly via altering neurotransmitter systems [29–31]; and (c) antidepressants damage normal cells, including osteoblasts, osteoclasts, hepatocytes, etc [25, 28]. These results suggest a possible iatrogenic pathway of antidepressant medication in inducing dementia. In addition, Gray et al. revealed that the cumulative use of tricyclic antidepressants, was related to a higher hazard ratio for dementia, and this finding was consistent with our results [52]. Lee et al. has recently published an article and also considered investigating the association between antidepressants and dementia. They concluded that SSRIs, MAOIs, and heterocyclic antidepressants were associated with an increased risk of dementia, which is consistent with our results. However, in their study, compared to non-dementia controls, dementia cases had longer follow up period from diagnosis of depression to endpoint of dementia. Therefore, higher odds of antidepressant usage in case group were predictable. Also, subjects that were diagnosed with dementia before antidepressant-prescribed date should be excluded because dementia would be more likely caused by other factors, such as depression status. In contrast to their study which focuses on only the depression patients, we had analyzed the interaction effect of depression status and antidepressant usage on the risk of dementia. In our study, case group and control group were followed from the same index date. Hence, our result should provide more generalized results and could be more widely applied to population regardless of the depression status [53].

Mossello et al. mentioned that antidepressant treatment would protect cognitive function in depressed older patients with AD [54]. They further suggested that cognitive protection was possibly via a reduction in depressive symptoms after taking antidepressants, while we proposed that antidepressant medication might increase the risk of dementia independent of depression. Another study using a nationwide database in Denmark revealed that continued long-term antidepressant treatment was associated with a reduced rate of dementia [55]. From a biological view of the treatment of depression, Sheline et al. proposed that Citalopram, an SSRI, decreased cerebrospinal fluid (CSF) amyloid beta protein production in a dose-dependent manner in both animal and human studies, and this ability may be a potential preventive strategy for AD [56].

Our conclusions being based on secondary data is the major limitation of this study. Therefore, we were unable to directly clarify the causality between the use of antidepressants and the development of dementia. To examine the temporal relationship between antidepressant medication and dementia, long-term clinical observational studies need to be carried out to further study this issue. Furthermore, the potential inaccuracies of clinical diagnosis and the use of ICD-9 codes in Taiwan with a study period from 2001 to 2011 are also the other limitations to this study. It is important to note that in Taiwan, diagnoses of dementia were made by neurologists or psychiatrists based on guidelines in DSM-IV or NINCDS-ADRDA Alzheimer's Criteria. In order to improve reliability of the results, the main outcome was confirmed by two repeated diagnoses of dementia.

In this study, we used Taiwan’s NHIRD to analyze the association between a series of antidepressant medications and the risk of dementia, and we set up a follow-up period of up to 9 years. (a) Both depression and non-depression case groups with antidepressants use had higher incidences of dementia and (b) high cDDDs of antidepressants were associated with increased risks of dementia. Together these lines of evidence reflect that antidepressant medications are associated with an increased risk for dementia in a manner independent of depression. Taiwan’s NHIRD is well-characterized with patients’ basic information, medical expenditures, and details of prescription records, so we could control for a number of potentially confounding factors, to provide more-accurate results. Thus, these reliable results indirectly suggest two possible pathways; (a) antidepressant medication increases the risk of dementia; and (b) it deteriorates the pathogenesis of dementia and further causes dementia symptoms to present earlier. Antidepressant medications are like a double-edged sword. They may exacerbate the risk of developing dementia, but importantly, they could also improve patients’ quality of life and working ability, and prevent suicide. More studies are needed to evaluate the comprehensive efficacy of antidepressant medication in patients.

## Conclusions

Multiple factors may participate in the formation of dementia, including age, smoking, stroke, depression, DM, hypertension, hyperlipidemia, anxiety, insomnia, etc. This study could not directly address whether taking antidepressants can induce dementia; however, we propose that antidepressant medication is associated with an increased risk of dementia. Different hazard ratios of the 6 classes of antidepressants in developing dementia further provide information about the relative drug safety for clinical use and suggest that caution should be exercised with the long-term use of antidepressants.

## Supporting information

S1 Fig The plot of log(-log(survival function)) versus log of survival time. (TIF)

S2 Fig Kaplan-Meier estimates of study and control group patients who developed dementia after 5 years of index date. (TIF)

## Abbreviations

NHIRD: National Health Insurance Research Database
HR: hazard ratio
CI: confidence interval
cDDDs: cumulative defined daily doses
MCI: mild cognitive impairment
VMD: very mild dementia
TCAs: tricyclic antidepressants
TeCAs: tetracyclic antidepressants
SSRIs: selective serotonin reuptake inhibitors
SARIs: serotonin antagonist and reuptake inhibitors
MAOIs: monoamine oxidase inhibitors
SNRIs: selective norepinephrine reuptake inhibitors
ICD-9-CM: International Classifications of Diseases; Ninth Revision; Clinical Modifications
ATC: Anatomical Therapeutic Chemical
DM: diabetes mellitus
DSM-IV: Diagnostic and Statistical Manual of Mental Disorders; Fourth Edition
IRB: Institutional Review Board
NT: New Taiwan
CCI: Charlson comorbidity index
IS: inescapable shock
CSF: cerebrospinal fluid
