# Supplementary figures and images for: Risk analysis of use of different classes of antidepressants on subsequent dementia: A nationwide cohort study in Taiwan
Source: PLoS One. 2017 Apr 6;12(4):e0175187. doi: 10.1371/journal.pone.0175187 (PMC5383251; doi:10.1371/journal.pone.0175187)

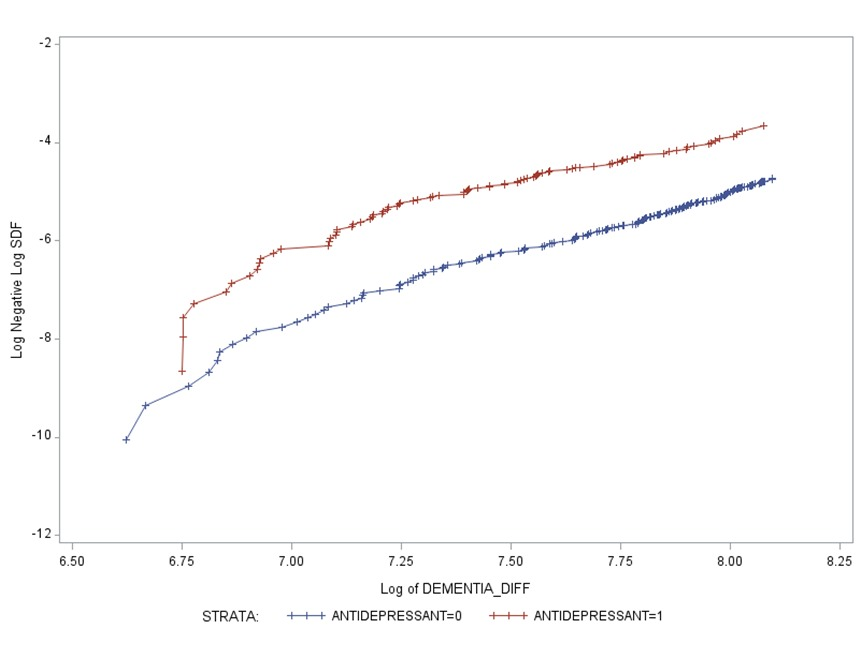

Supplement: S1 Fig — (TIF) [file pone.0175187.s001.tif]

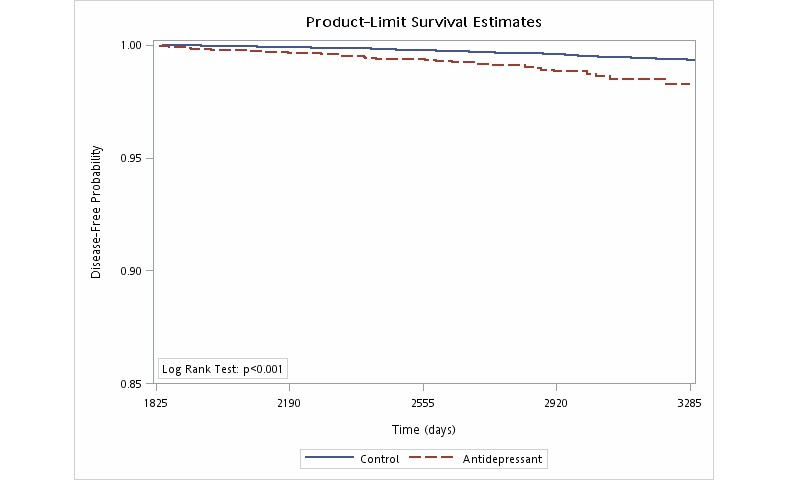

Supplement: S2 Fig — (TIF) [file pone.0175187.s002.tif]
